# Dataset on biochemical inhibiting activities of selected phytochemicals in *Azadirachta indica L* as potential NS2B–NS3 proteases inhibitors

**DOI:** 10.1016/j.dib.2023.109162

**Published:** 2023-04-18

**Authors:** Abel Kolawole Oyebamiji, Sunday A. Akintelu, Ismail O. Akande, Halleluyah O. Aworinde, Oluwafunmilola A. Adepegba, Emmanuel T. Akintayo, Cecillia O. Akintayo, Banjo Semire, Jonathan O. Babalola

**Affiliations:** a Industrial Chemistry Programme, Bowen University, PMB 284, Iwo, Osun State, Nigeria; b Department of Pure and Applied Chemistry, Ladoke Akintola University of Technology, P.M.B. 4000, Ogbomoso, Oyo State, Nigeria; c Department of Basic Sciences, Adeleke University, P.M.B. 250, Ede, Osun State, Nigeria; d College of Computing and Communication Studies, Bowen University, Iwo, Nigeria; e Department of Chemistry, University of Ibadan, Ibadan, Oyo State, Nigeria

**Keywords:** *Azadirachta indica* L., NS2B–NS3 proteases, Dengue, Virus, DFT, Affinity

## Abstract

The anti-NS2B–NS3 proteases activities of *Azadirachta indica* L. were investigated via the data obtained from selected bioactive compounds from *Azadirachta indica* L. The work was investigated using *insilico* approach and the series of computational software were used to execute the task. The software used were Spartan 14, material studio, Padel, Pymol, Autodock tool, Autodock vina and discovery studio. The obtained descriptors from 2D and 3D of the optimized compounds were screened and they were used to develop QSAR model using material studio software. Also, biological interaction between the selected bioactive compounds from *Azadirachta indica* L. and NS2B–NS3 proteases (PDB ID: 2fom) were accomplished using docking method and the calculated binding affinity as well as the residues involved in the interaction were reported. More so, the ADMET features for [(5*S*,6*R*,7*S*,8*R*,9*S*,10*R*,11*S*,12*R*,13*S*,17*R*)-17-(2,5-dihydroxy-2,5-dihydrofuran-3-yl)-11,12-dihydroxy-6‑methoxy-4,4,8,10,13-pentamethyl-1,16-dioxo-6,7,9,11,12,17-hexahydro-5*H*-cyclopenta[*a*]phenanthren-7-yl] 3-methylbut-2-enoate (**Compound 6)** and (10*R*,13*S*,14*S*,17*S*)-17-[1-(3,4-dihydroxy-5,5-dimethyloxolan-2-yl)ethyl]-4,4,10,13,14-pentamethyl-1,2,5,6,9,11,12,15,16,17-decahydrocyclopenta[a]phenanthren-3-one (**compound 12**) with lowest binding affinity were investigated and reported.

**Specification Table**

**Table:** 

Subject	Insilico Study
Specific subject area	Drug Discovery and Design
Type of data	Figure Table Binding Affinity Statistical model
How data were acquired	Spartan’14, PADEL, Pymol 1.7.4.4, Autodock tool 1.5.6; AutoVina, Discovery studio
Data format	Raw figures
Description of data collection	The initial step for this work was selection of appropriate compound structures for optimization using 6-31G** basis set via density functional theory method. The data obtained that described the activities of the selected compounds were employed in developing QSAR model via Genetic function Algorithm using material studio software. The selected compounds from *Azadirachta indica* L. were docked against NS2B–NS3 proteases (PDB ID: 2fom) using docking methods and the outputs were subjected to interpretation.
Data source location	Computational Chemistry Research Laboratory, Department of Chemistry and Industrial Chemistry, BOWEN University, Iwo, Osun State, Nigeria
Data accessibility	The experimental and predicted data can be accessed in the data article (https://data.mendeley.com/datasets/6br4x267tf/1); DOI:10.17632/6br4x267tf.1

## Value of the Data

• The data generated from the optimized selected phytochemicals from *Azadirachta indica* L. will help researchers to have good understanding of anti- NS2B–NS3 Proteases of the examined structures.
• The developed quantitative structure-activities relationship (QSAR) model using data obtained from 2D and 3D structurers of selected phytochemicals from *Azadirachta indica* L. will help researchers in recommending collection of compounds with efficient biological interactions present in *Azadirachta indica* L.
• The calculated descriptors (2D and 3D) obtained from optimized structures of selected phytochemicals from *Azadirachta indica* L. will expose scientists to the type of descriptors with perfect description of anti- NS2B–NS3 Proteases of the examined structures.
• The scoring value of individual investigated phytochemical will disclose to scientists which of the compound has greater ability to acts as NS2B–NS3 Proteases (PDB ID: 2fom) inhibitor
• The absorption, distribution, metabolism, excretion, and toxicity (ADMET) capacity of the investigated phytochemicals will help scientists to know the possible operation of each compound in human body.

## 1 Data Description

Table 1 revealed the 3-dimensional structures of phytochemicals present in *Azadirachta indica*. L obtained from the work carried out by Saleem et al., 2018 [1]. The compounds were optimized using B3LYP with 6–31G** as basis set.

**Table 1:** 3-dimensional structures of selected phytochemicals in *Azadirachta indica* L.

	3D Structure	IUPAC Name
1		1-methyl-4-propan-2-ylbenzene
2*		[(5*R*,6*R*,7*S*,8*R*,9*R*,10*R*,13*S*,17*R*)−17-(2,3-dihydrofuran-4-yl)−6‑hydroxy-4,4,8,10,13-pentamethyl-3-oxo-5,6,7,9,11,12,16,17-octahydrocyclopenta[*a*]phenanthren-7-yl] acetate
3		1-methyl-4-propan-2-ylcyclohexa-1,3-diene
4		[(5*R*,6*R*,8*R*,9*R*,10*R*,13*S*,17*R*)−17-(furan-3-yl)−6‑hydroxy-4,4,8,10,13-pentamethyl-3-oxo-1,2,5,6,7,9,11,12,16,17-decahydrocyclopenta[*a*]phenanthren-7-yl] acetate
5		[(1*S*,4*bR*,5*R*,6*aR*,10*aR*,10*bR*,12*aR*)−1-[(*E*)‑*hex*-2-*en*-2-*yl*]−1‑methoxy-4*b*,7,7,10*a*,12*a*-pentamethyl-3,8-dioxo-5,6,6*a*,10*b*,11,12-hexahydronaphtho[2,1-*f*]isochromen-5-yl] acetate
6		[(5*S*,6*R*,7*S*,8*R*,9*S*,10*R*,11*S*,12*R*,13*S*,17*R*)−17-(2,5-dihydroxy-2,5-dihydrofuran-3-yl)−11,12-dihydroxy-6‑methoxy-4,4,8,10,13-pentamethyl-1,16-dioxo-6,7,9,11,12,17-hexahydro-5*H*-cyclopenta[*a*]phenanthren-7-yl] 3-methylbut-2-enoate
7		(4*aS*,10*aS*)−6‑hydroxy-1,1,4*a*,7-tetramethyl-3,4,10,10*a*-tetrahydro-2*H*-phenanthren-9-one
8*		4-[(3*E*,5*E*,7*E*)−8,10-*dimethyl*-2-*methylideneundeca*-3,5,7,9-*tetraenyl*]−2-methylbenzene-1,3-dicarboxylic acid
9		[(5*R*,6*R*,7*S*,9*R*,10*R*,13*S*,17*R*)−17-(furan-3-yl)−6‑hydroxy-4,4,8,10,13-pentamethyl-3-oxo-5,6,7,9,11,12,16,17-octahydrocyclopenta[*a*]phenanthren-7-yl] acetate
10		[(5*R*,6*R*,7*S*,8*R*,9*R*,10*R*,13*S*,17*R*)−17-(furan-3-yl)−6‑hydroxy-4,4,8,10,13-pentamethyl-3-oxo-5,6,7,9,11,12,16,17-octahydrocyclopenta[*a*]phenanthren-7-yl] acetate
11		tridecylbenzene
12		(10*R*,13*S*,14*S*,17*S*)−17-[1-(3,4-*dihydroxy*-5,5-*dimethyloxolan*-2-*yl*)*ethyl*]−4,4,10,13,14-pentamethyl-1,2,5,6,9,11,12,15,16,17-decahydrocyclopenta[*a*]phenanthren-3-one
13		(3*S*,8*S*,9*S*,10*R*,13*R*,14*S*,17*R*)−17-[(*E*,2*R*,5*S*)−5-*ethyl*-6-*methylhept*-3-*en*-2-*yl*]−10,13-dimethyl-2,3,4,7,8,9,11,12,14,15,16,17-dodecahydro-1*H*-cyclopenta[*a*]phenanthren-3-ol
14		(4*aS*,10*aS*)−6‑hydroxy-1,1,4*a*-trimethyl-7-propan-2-yl-3,4,10,10*a*-tetrahydro-2*H*-phenanthren-9-one
15		(1*S*)−4-methyl-1-propan-2-ylcyclohex-3-en-1-ol
16*		[(6*R*,13*S*)−6‑methoxy-4,4,8,10,13-pentamethyl-3,16-dioxo-17-[(2*S*)−1-*oxobutan*-2-*yl*]−6,7,9,11,12,17-hexahydro-5*H*-cyclopenta[*a*]phenanthren-7-yl] acetate

⁎ denotes test set.

Tables 2 and 3 showed the calculated descriptors from 3D and 2D structures respectively. The descriptors which describe the anti- NS2B–NS3 Proteases activities of phytochemicals from *Azadirachta indica*. L were examined and screened. The descriptors from 3D structures of selected phytochemicals from *Azadirachta indica*. L were highest occupied molecular orbital energy (E_HOMO_), lowest unoccupied molecular orbital energy (E_LUMO_), band-gap, dipole moment, weight, area, polar surface area, log P, hydrogen bond donor (HBD) and hydrogen bond acceptor (HBA) while the descriptors from 2D descriptors were ALogP, ALogP2, AMR, Apol, nATOM, nHeavyAtom, ATS0m, ATS1m, ATS2m, ATS3m, ATS4m.

**Table 2:** Calculated descriptors for 3D structures of selected phytochemicals from *Azadirachta indica* L.

	E_HOMO_	E_LUMO_	BG	DM	MW	AREA	VOL	PSA	OVA	LOG P	POL	HBD	HBA
1	−6.14	**−0.11**	6.03	0.10	134.22	192.66	171.98	0.00	1.29	2.65	53.85	0	0
2	−5.23	−2.06	3.17	5.12	454.70	439.77	472.27	57.67	1.50	4.32	78.93	1	4
3	−5.16	−0.28	4.88	0.60	136.23	196.86	176.33	0.00	1.29	2.96	54.52	0	0
4	−5.97	−0.43	5.54	3.76	454.60	460.67	478.94	57.84	1.56	0.00	78.94	1	4
5	−6.51	−1.49	5.02	6.51	512.68	534.95	548.69	60.02	1.65	7.18	84.70	0	4
6	−5.24	−2.24	3.00	9.12	586.67	528.24	571.30	120.59	1.59	3.42	86.97	4	9
7	−6.07	−1.09	4.98	2.65	272.38	300.19	299.33	32.71	1.39	2.99	64.48	1	2
8	−5.05	−1.43	3.62	2.30	366.45	436.01	405.90	66.88	1.64	3.54	73.45	2	2
9	−5.27	−2.07	3.20	5.17	452.59	435.40	468.59	56.93	1.49	0.00	78.63	1	4
10	−5.27	−2.07	3.20	5.17	452.59	435.40	468.59	56.93	1.49	0.00	78.63	1	4
11	−6.34	0.14	6.48	0.41	260.46	375.31	337.17	0.00	1.60	6.73	67.19	0	0
12	−5.99	−0.29	5.70	4.07	472.71	496.95	518.41	55.63	1.59	5.72	82.08	2	4
13	−6.11	0.72	6.83	1.83	412.70	492.42	490.58	19.81	1.64	7.82	79.55	1	1
14	−6.09	−1.12	4.97	4.63	300.44	338.04	336.09	32.20	1.45	3.74	67.46	1	2
15	−6.32	0.77	7.09	1.58	154.25	206.74	187.79	17.45	1.30	2.23	54.93	1	1
16	−6.41	−1.49	4.92	5.52	484.63	485.61	509.45	66.89	1.57	4.67	81.47	0	5

**Table 3:** Calculated descriptors for 2D structures of selected phytochemicals from *Azadirachta indica* L.

AMR	apol	nAtom	nHeavyAtom	nH	nC	ATS0m	ATS1m	ATS2m	ATS3m
128.402	78.62813	71	33	38	28	5357.846	6137.161	10,329.19	13,158.34
19.7699	26.9351	24	10	14	10	1456.866	1612.14	2126.72	1982.461
47.6977	28.26869	26	10	16	10	1458.898	1636.355	2177.18	2058.151
110.0689	78.62813	71	33	38	28	5357.846	6137.161	10,325.17	13,182.55
142.8186	88.71089	81	37	44	31	6052.703	6734.376	11,254.79	15,221.54
153.6901	92.34531	84	42	42	32	7218.807	7783.425	13,161.21	17,358.94
42.7917	54.77738	51	19	32	19	2773.532	3128.445	3639.056	3414.368
56.1313	49.28703	44	20	24	18	3133.076	3564.2	5837.22	7168.801
88.8093	61.02462	53	27	26	23	4368.364	4409.555	6058.596	6628.995
110.8978	77.29455	69	33	36	28	5355.814	6112.947	10,274.71	13,090.74
110.8978	77.29455	69	33	36	28	5355.814	6112.947	10,274.71	13,090.74
135.729	88.01406	82	34	48	30	5400.567	6310.716	11,024.13	14,438.13
125.2911	83.84806	78	30	48	29	4488.399	5393.776	8513.826	10,394.49
64.897	55.4742	50	22	28	20	3425.668	3901.157	6345.703	7848.811
47.9186	30.40427	29	11	18	10	1716.898	1856.753	2791.01	2981.101
133.0031	82.52372	75	35	40	29	5760.11	6349.519	10,529.43	14,340.61

Fig. 1 revealed the orbital energy from 3D structure of selected phytochemicals present in *Azadirachta indica*. L. The E_HOMO_ and E_LUMO_ value on the energy profile was measured in eV (Fig. 2, Fig. 3, Fig. 4, Fig. 5, Fig. 6, Fig. 7, Fig. 8, Fig. 9, Fig. 10, Fig. 11, Fig. 12, Fig. 13, Fig. 14, Fig. 15, Fig. 16). Also, the actual difference between the LUMO energy and HOMO energy were clearly shown on Fig. 1, Fig. 2, Fig. 3, Fig. 4, Fig. 5, Fig. 6, Fig. 7, Fig. 8, Fig. 9, Fig. 10, Fig. 11, Fig. 12, Fig. 13, Fig. 14, Fig. 15, Fig. 16. More so, Table 4 showed HOMO-LUMO overlay of the investigated selected phytochemicals.

**Fig. 1:**
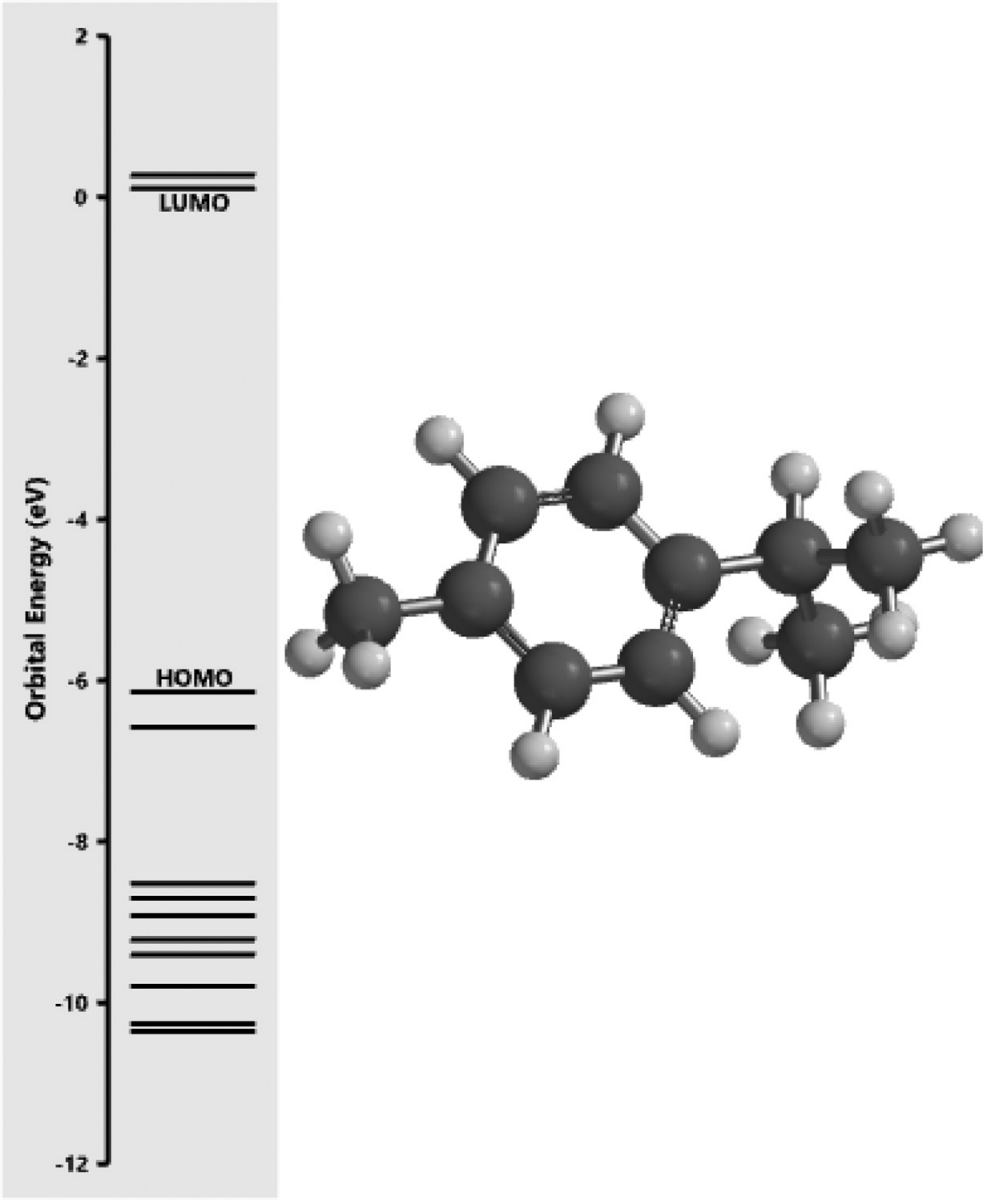
Predicted orbital energy for compound 1.

**Fig. 2:**
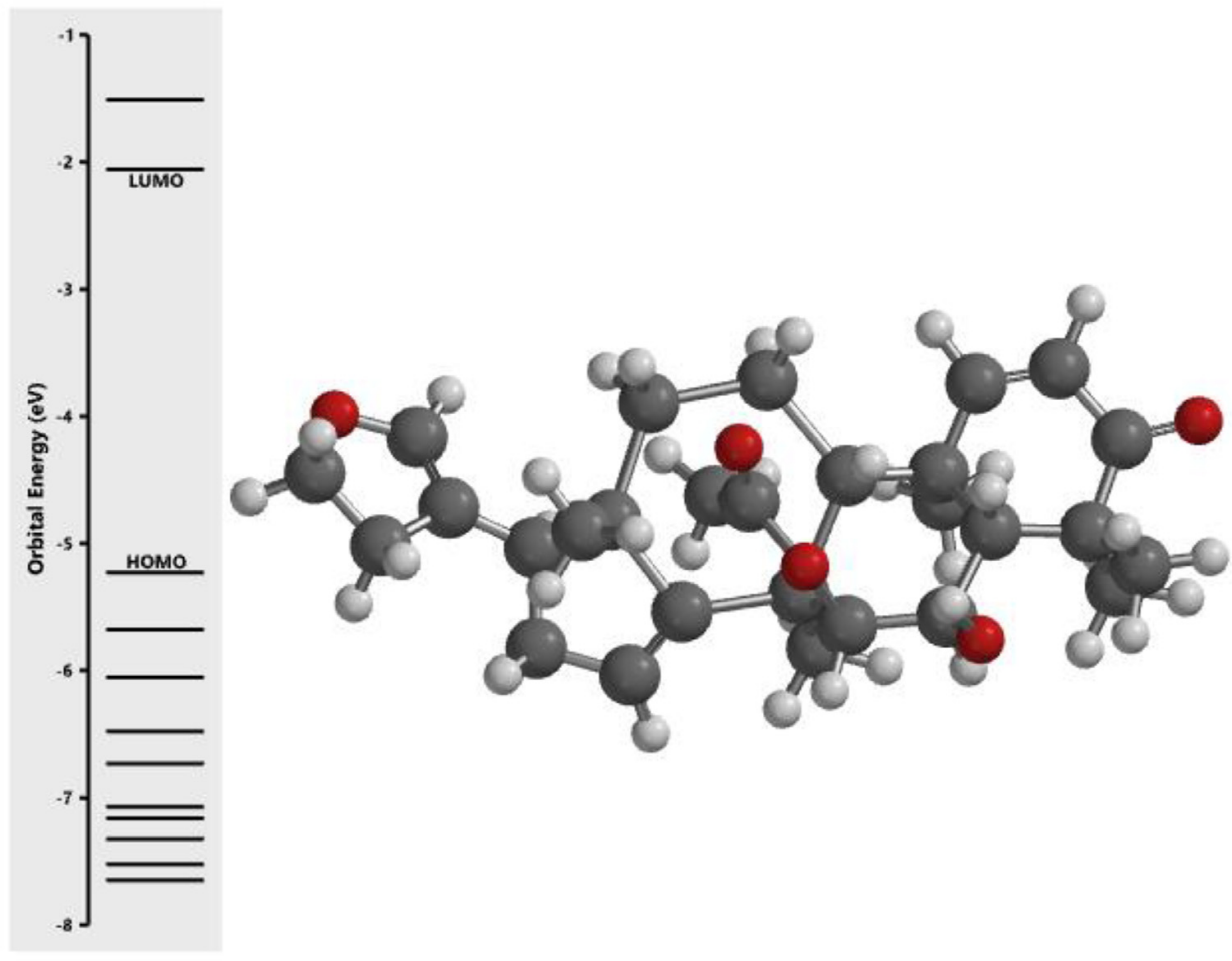
Predicted orbital energy for compound 2.

**Fig. 3:**
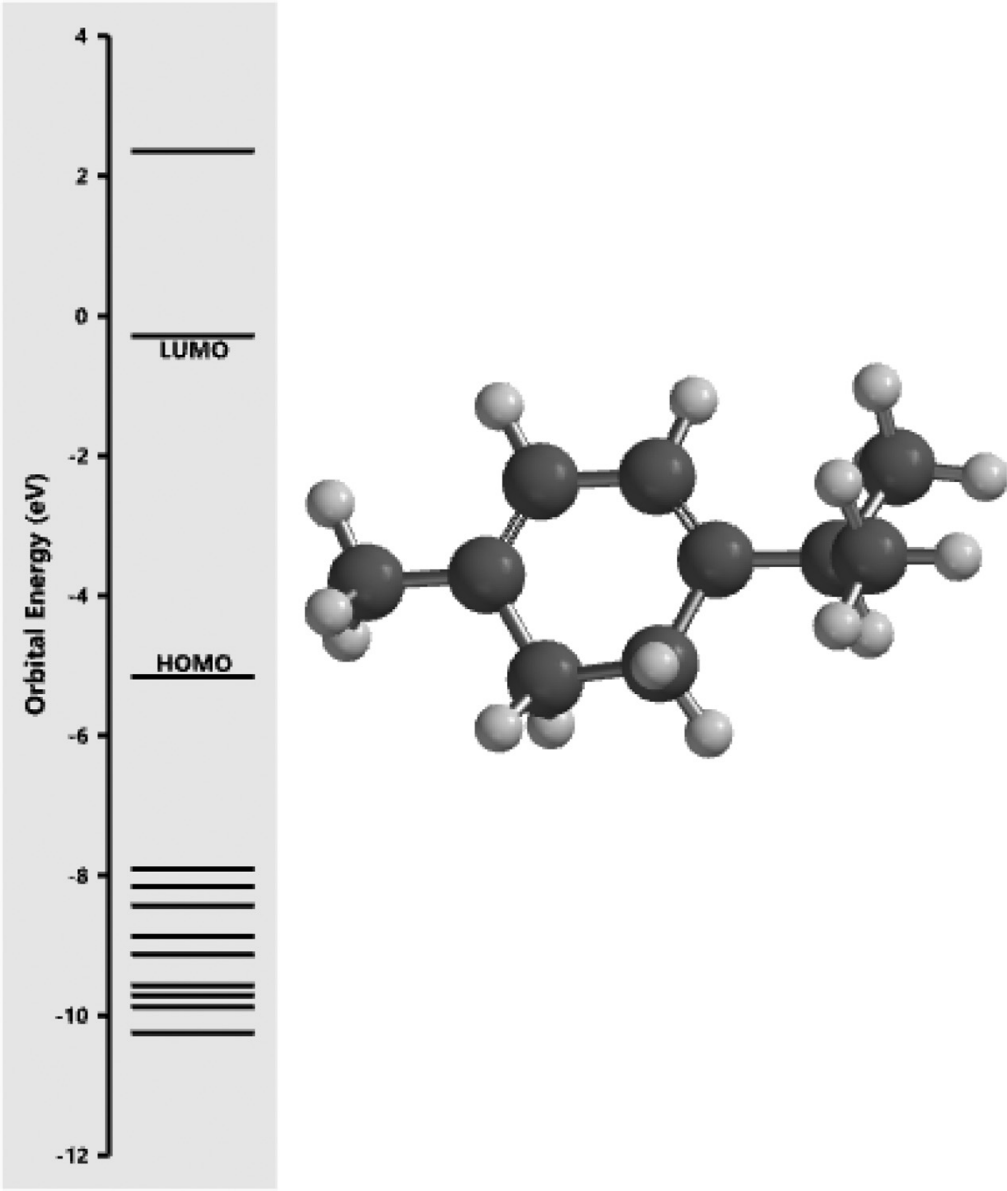
Predicted orbital energy for compound 3.

**Fig. 4:**
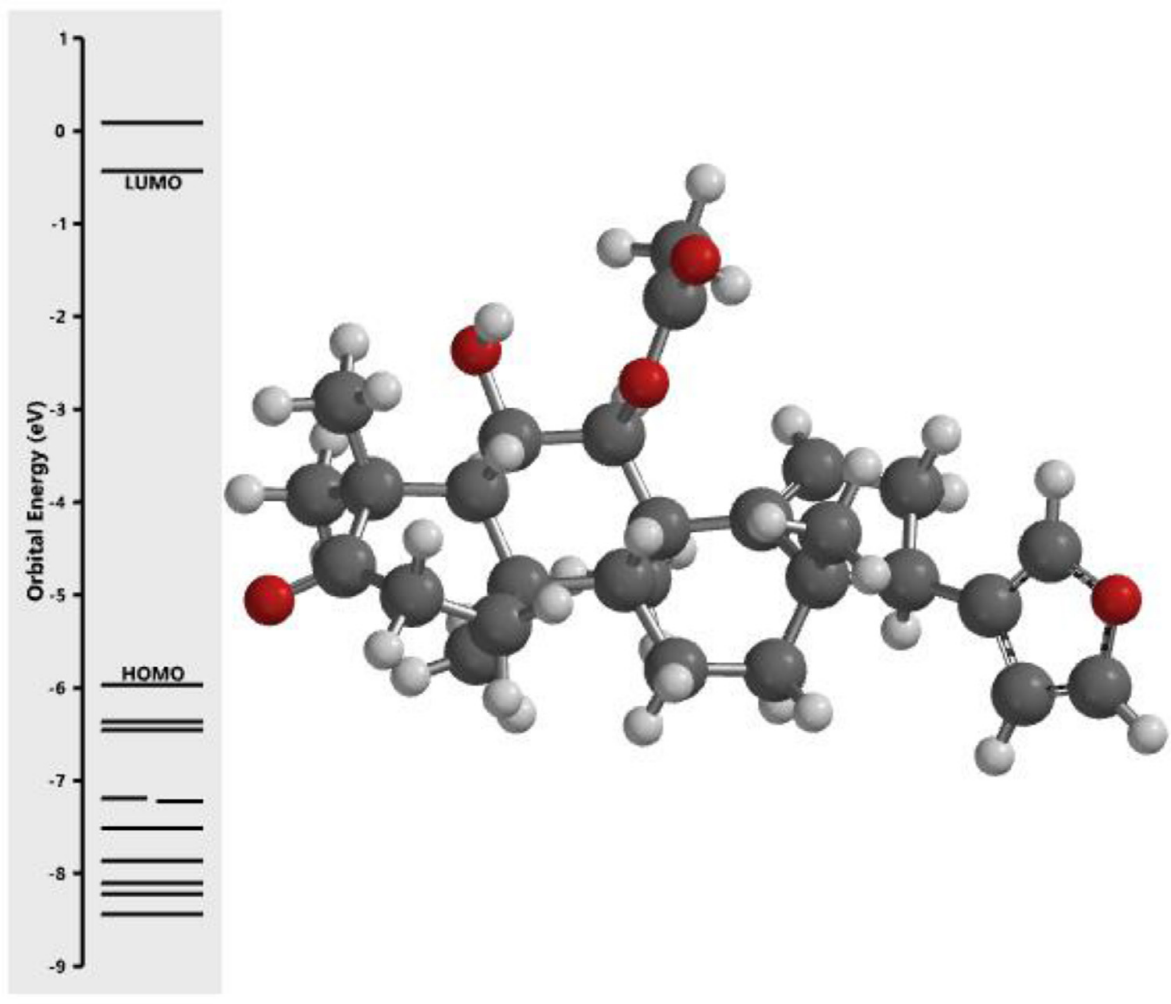
Predicted orbital energy for compound 4.

**Fig. 5:**
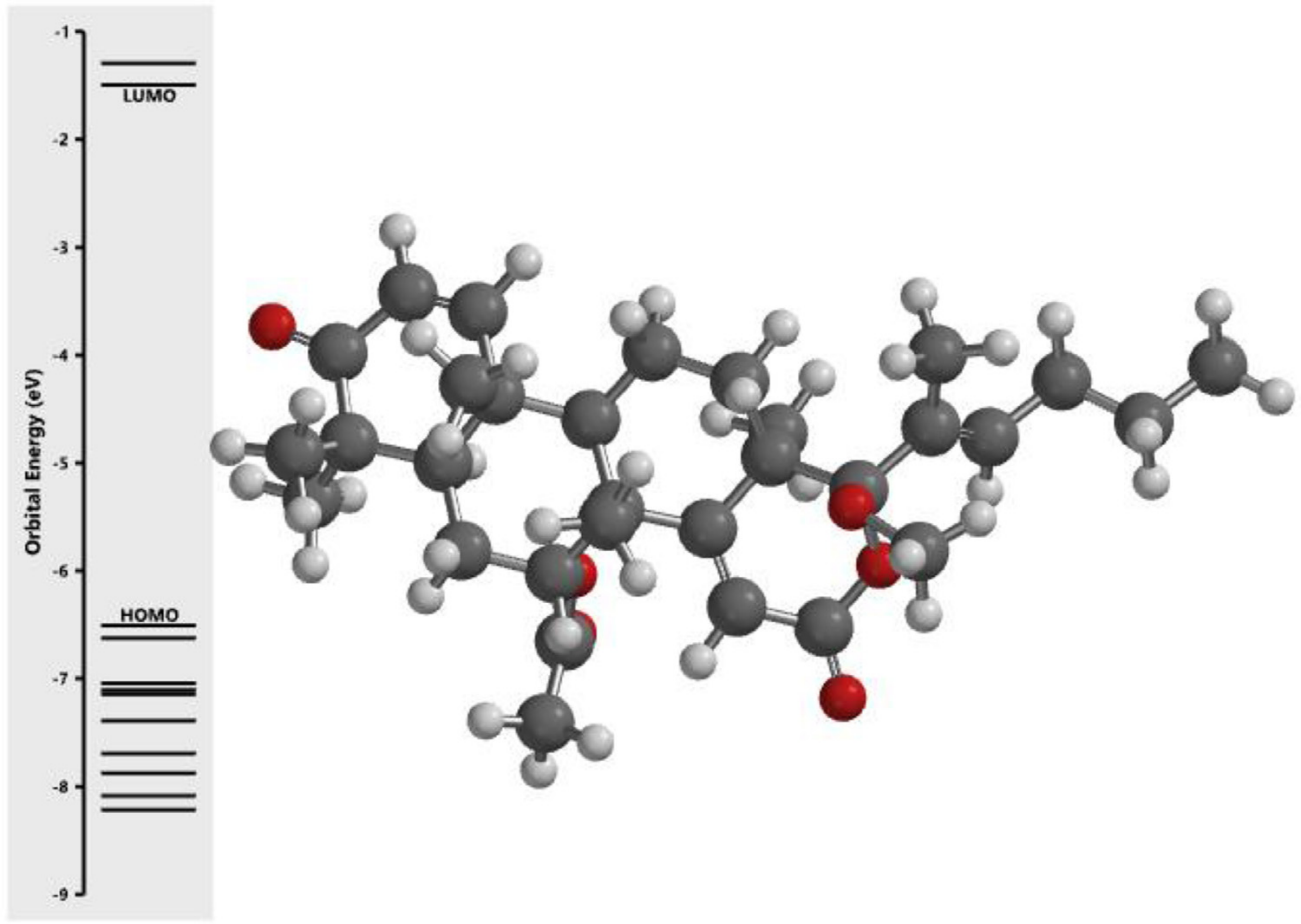
Predicted orbital energy for compound 5.

**Fig. 6:**
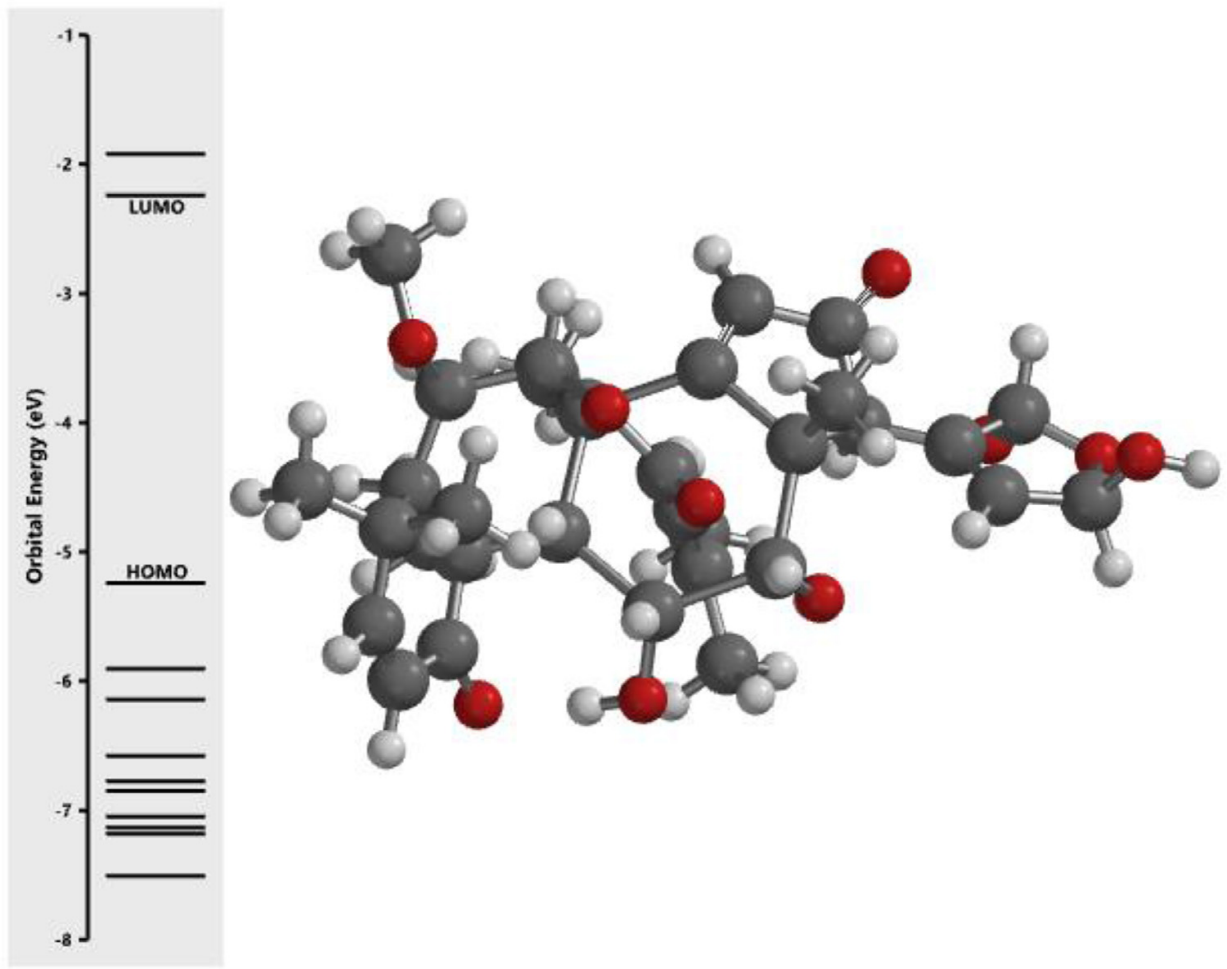
Predicted orbital energy for compound 6.

**Fig. 7:**
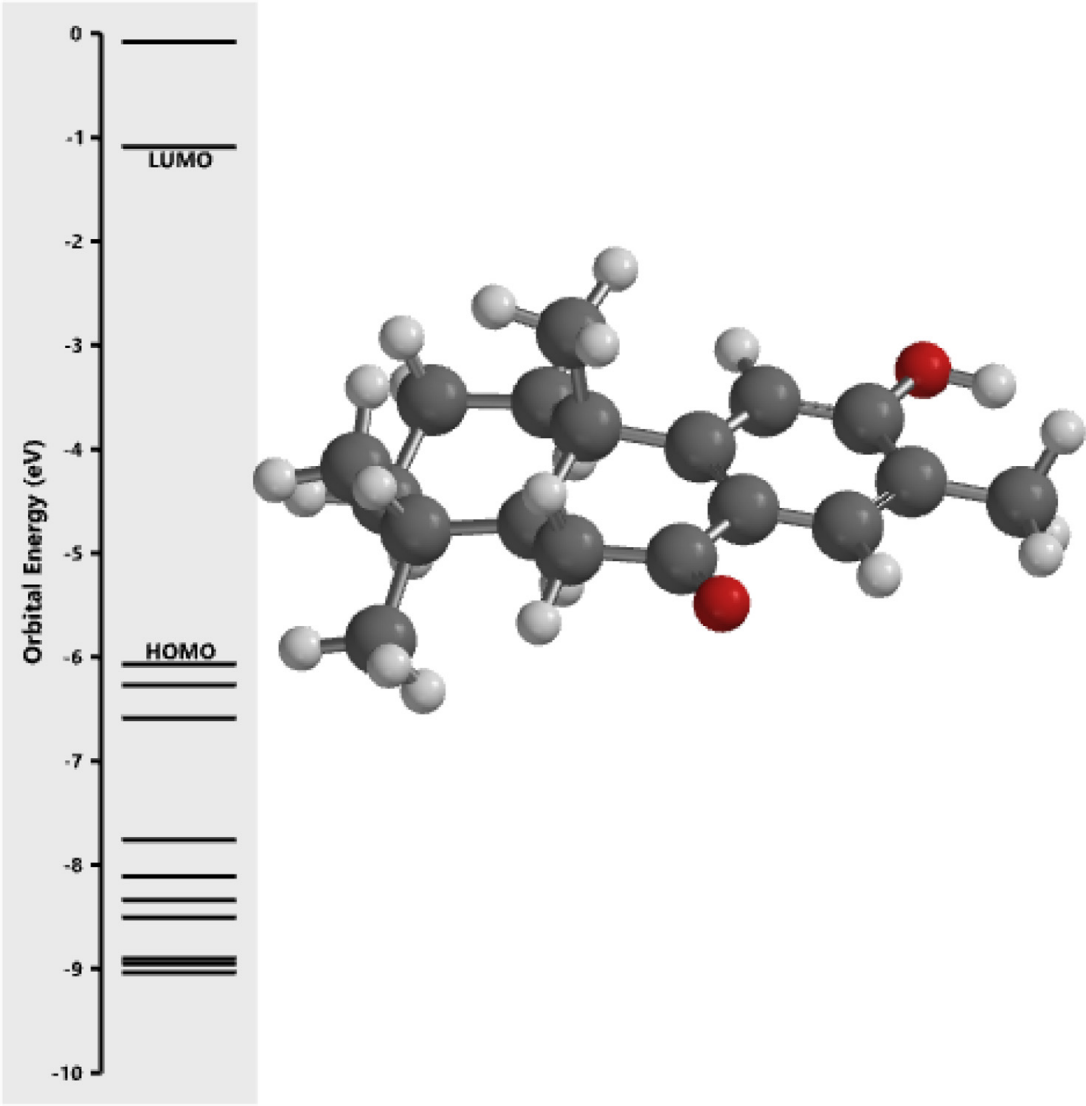
Predicted orbital energy for compound 7.

**Fig. 8:**
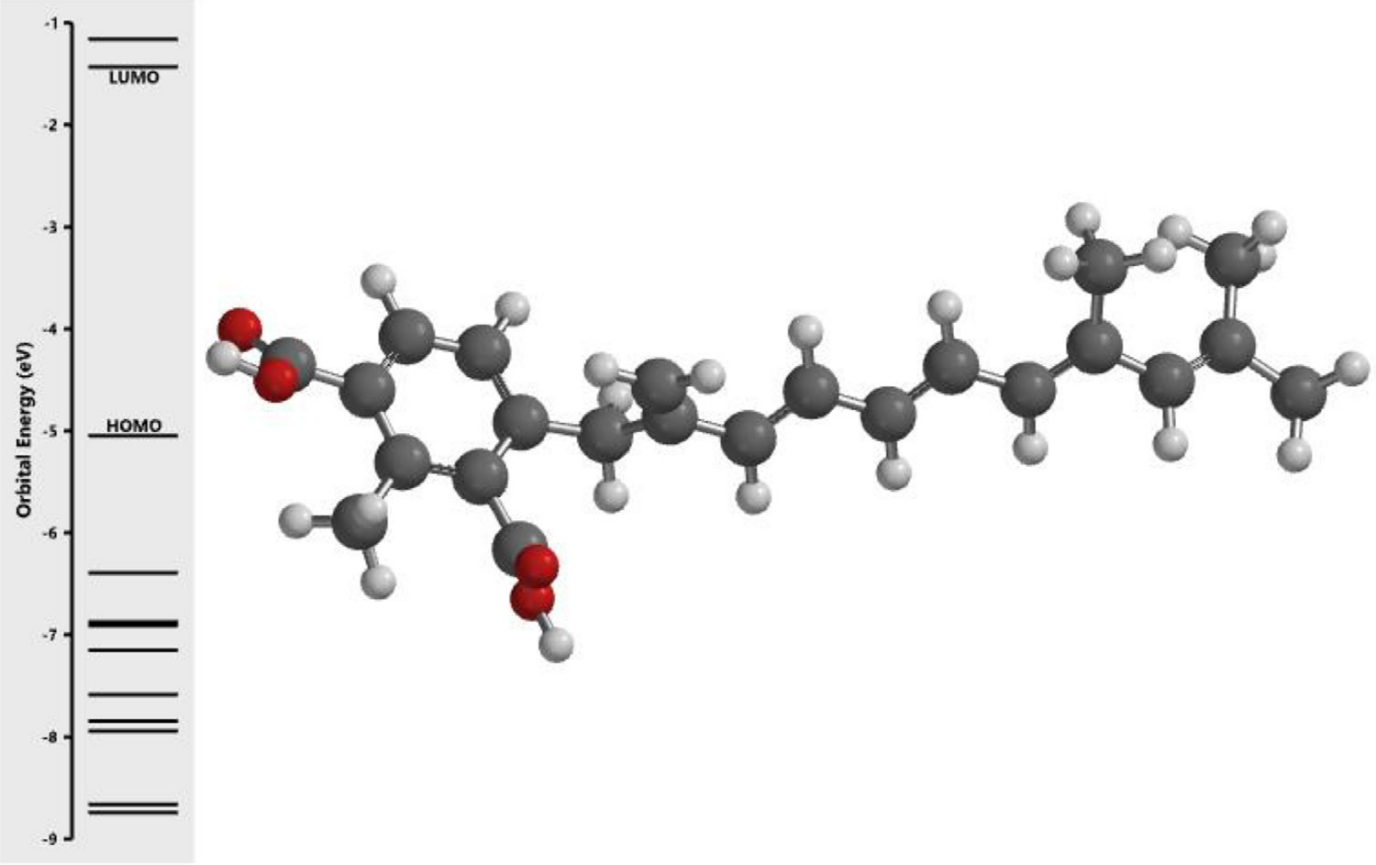
Predicted orbital energy for compound 8.

**Fig. 9:**
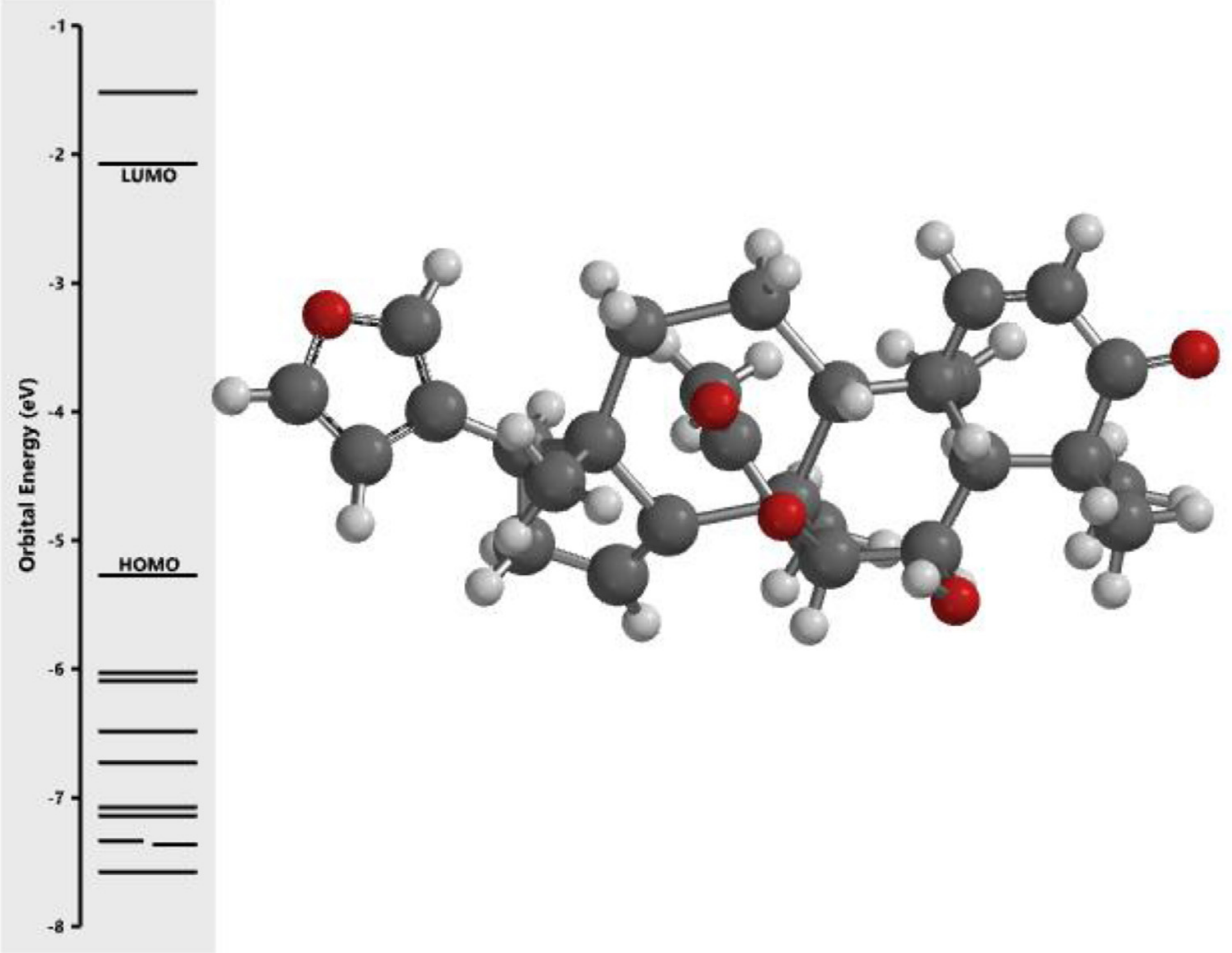
Predicted orbital energy for compound 9.

**Fig. 10:**
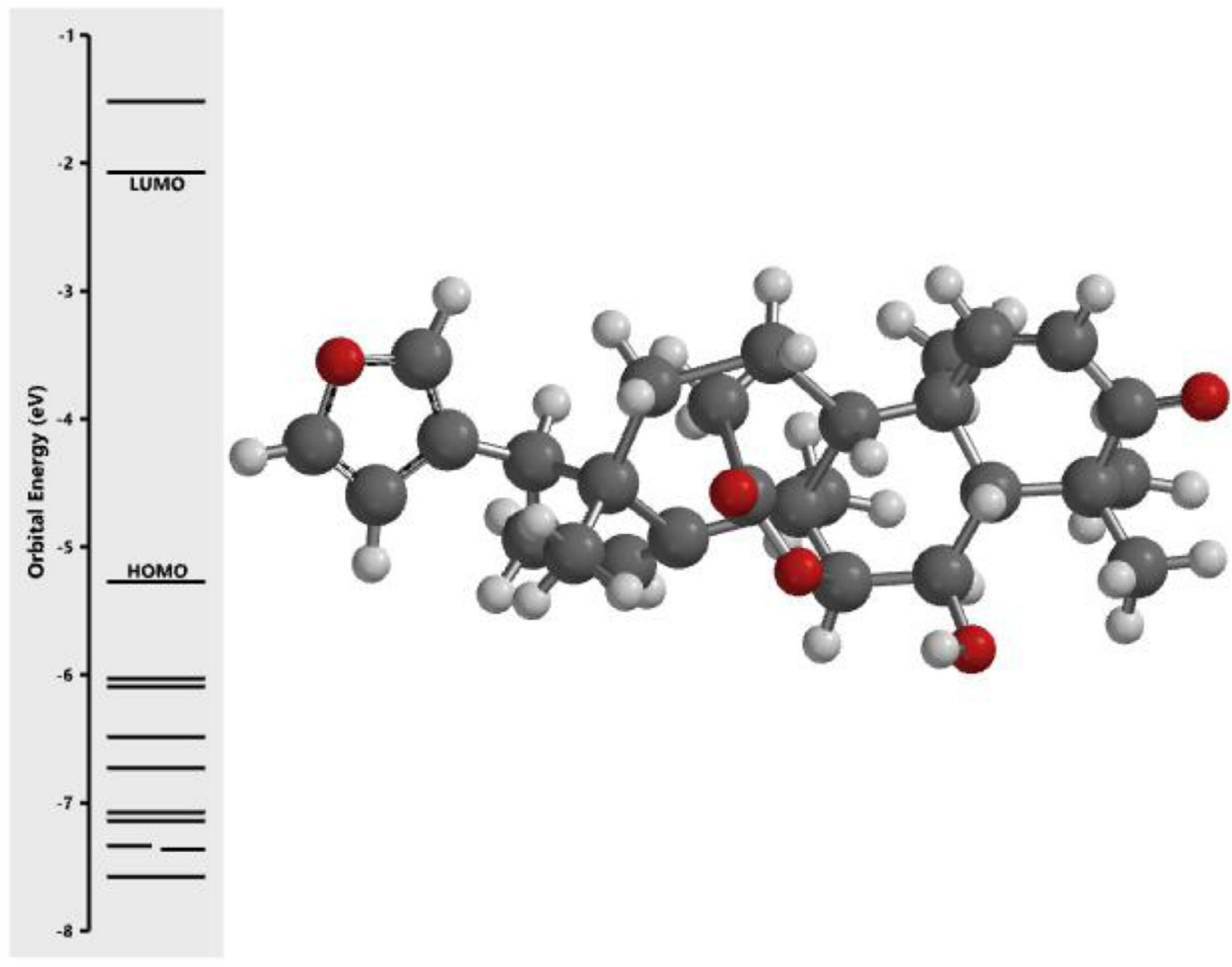
Predicted orbital energy for compound 10.

**Fig. 11:**
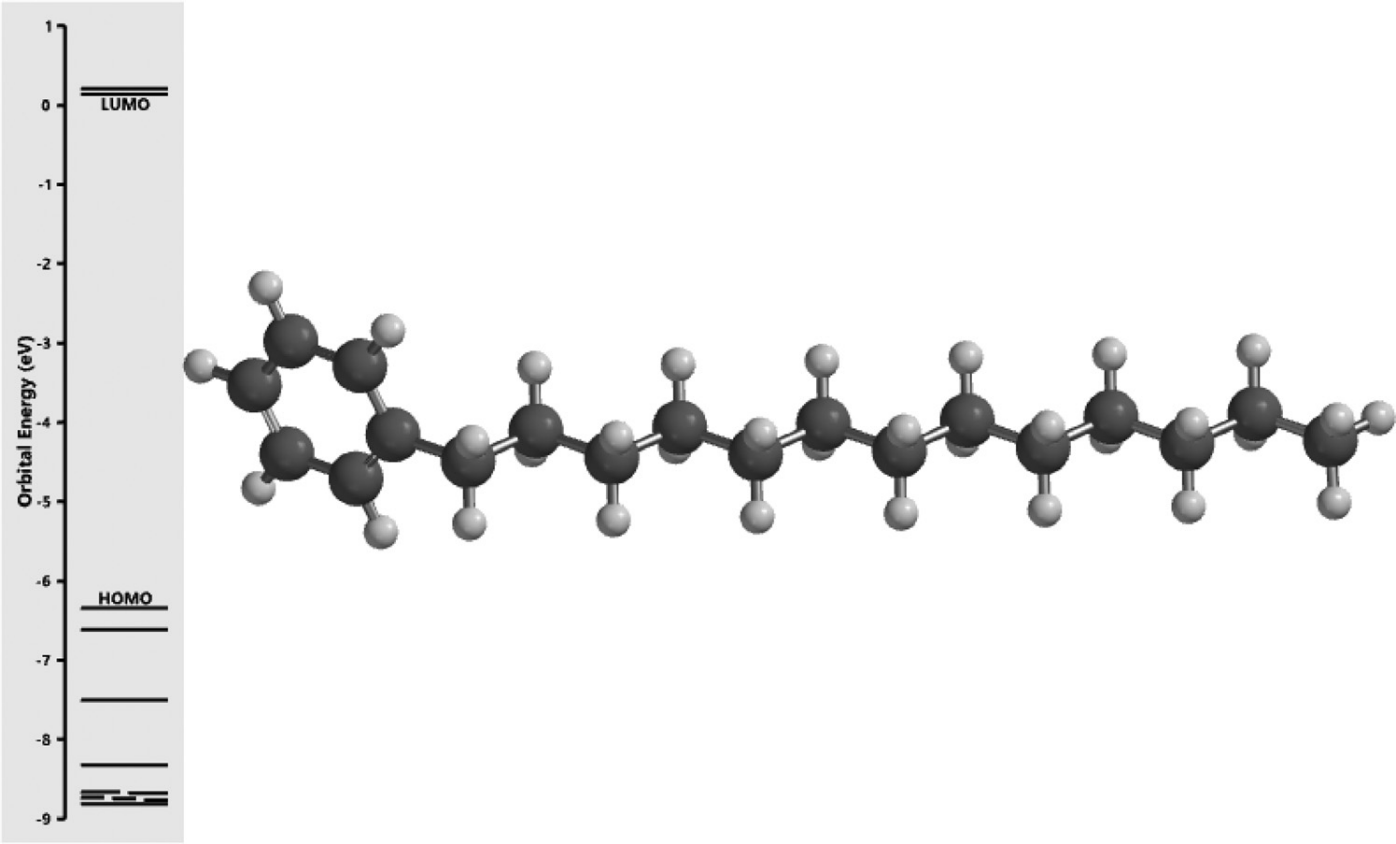
Predicted orbital energy for compound 11.

**Fig. 12:**
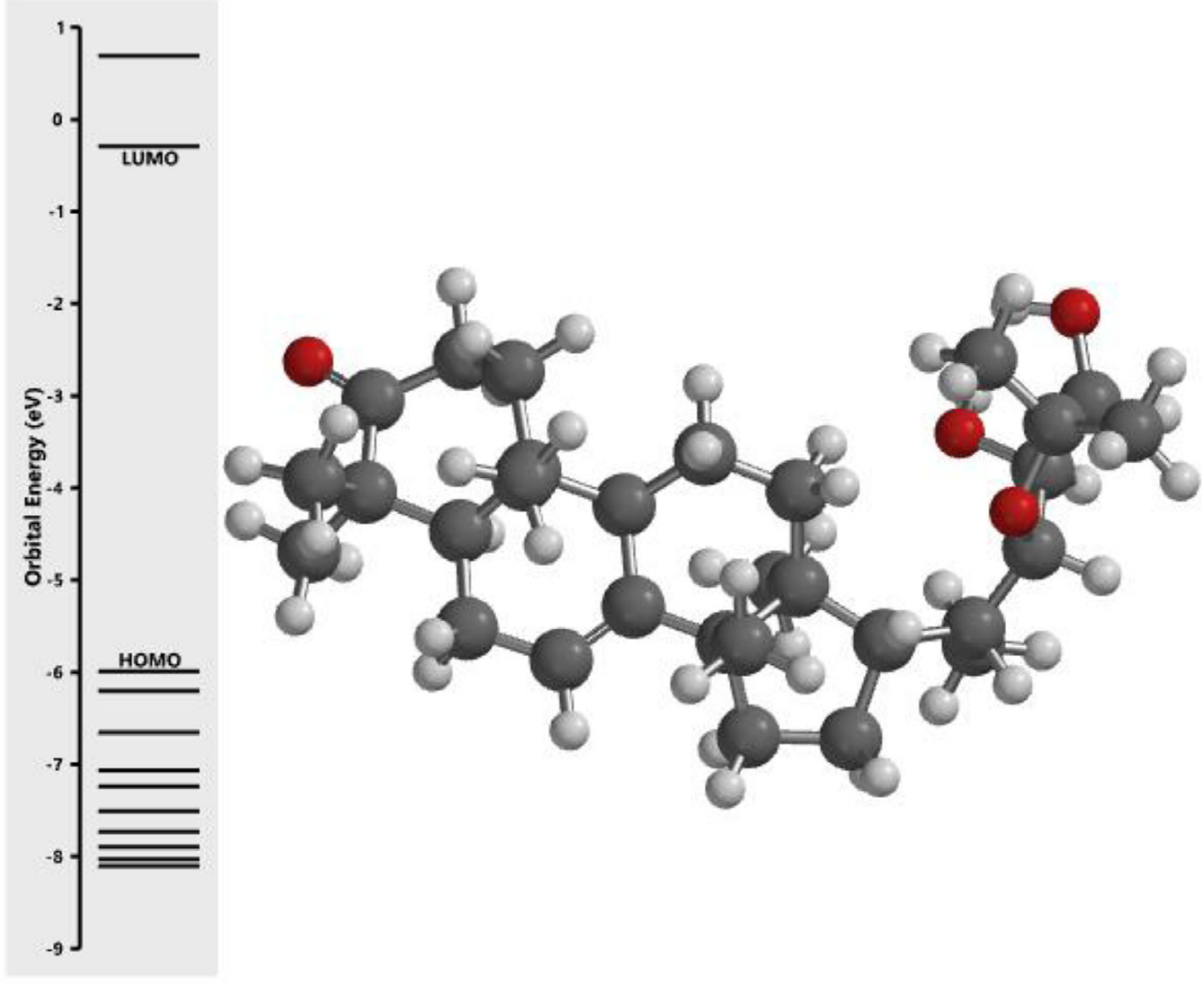
Predicted orbital energy for compound 12.

**Fig. 13:**
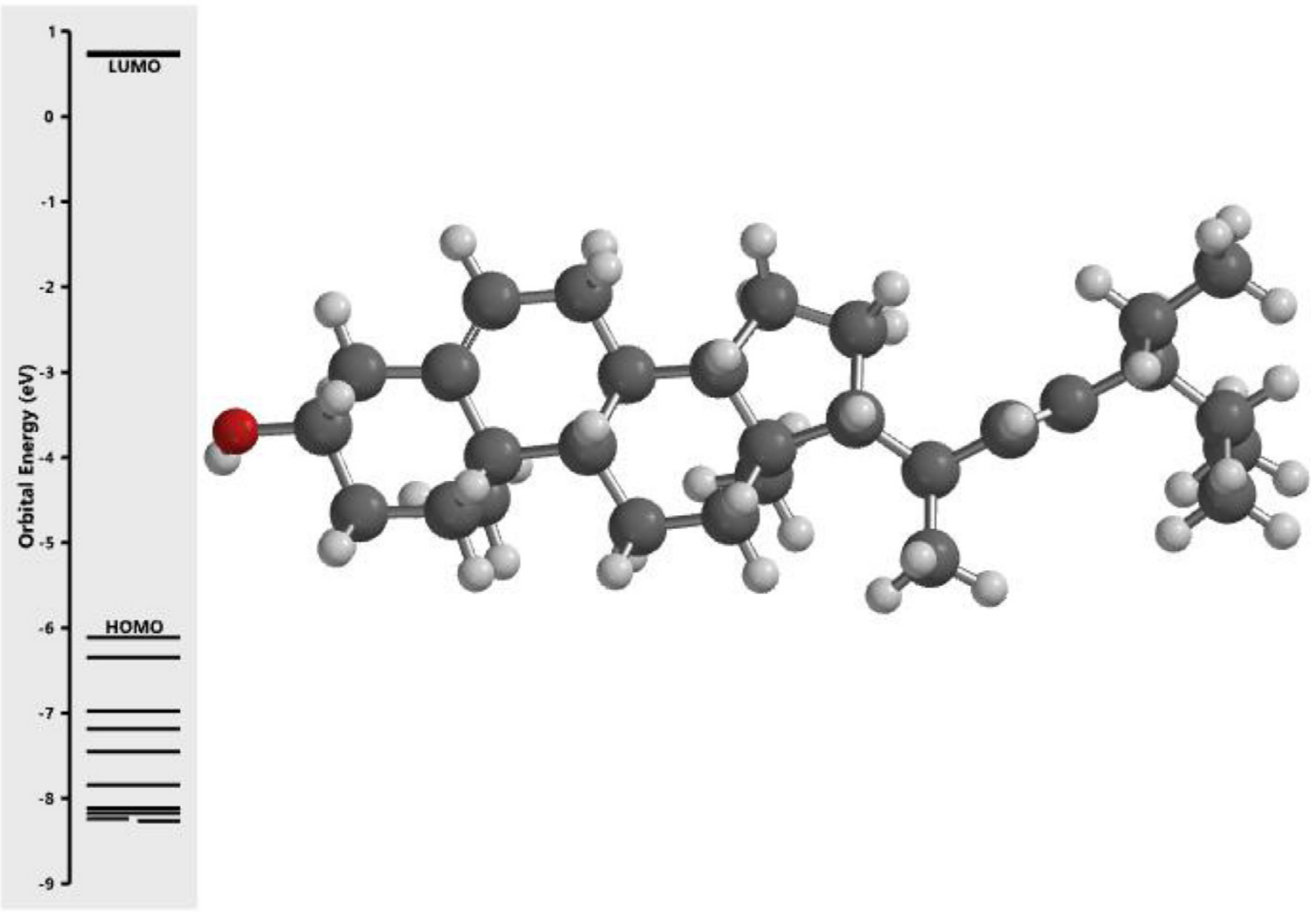
Predicted orbital energy for compound 13.

**Fig. 14:**
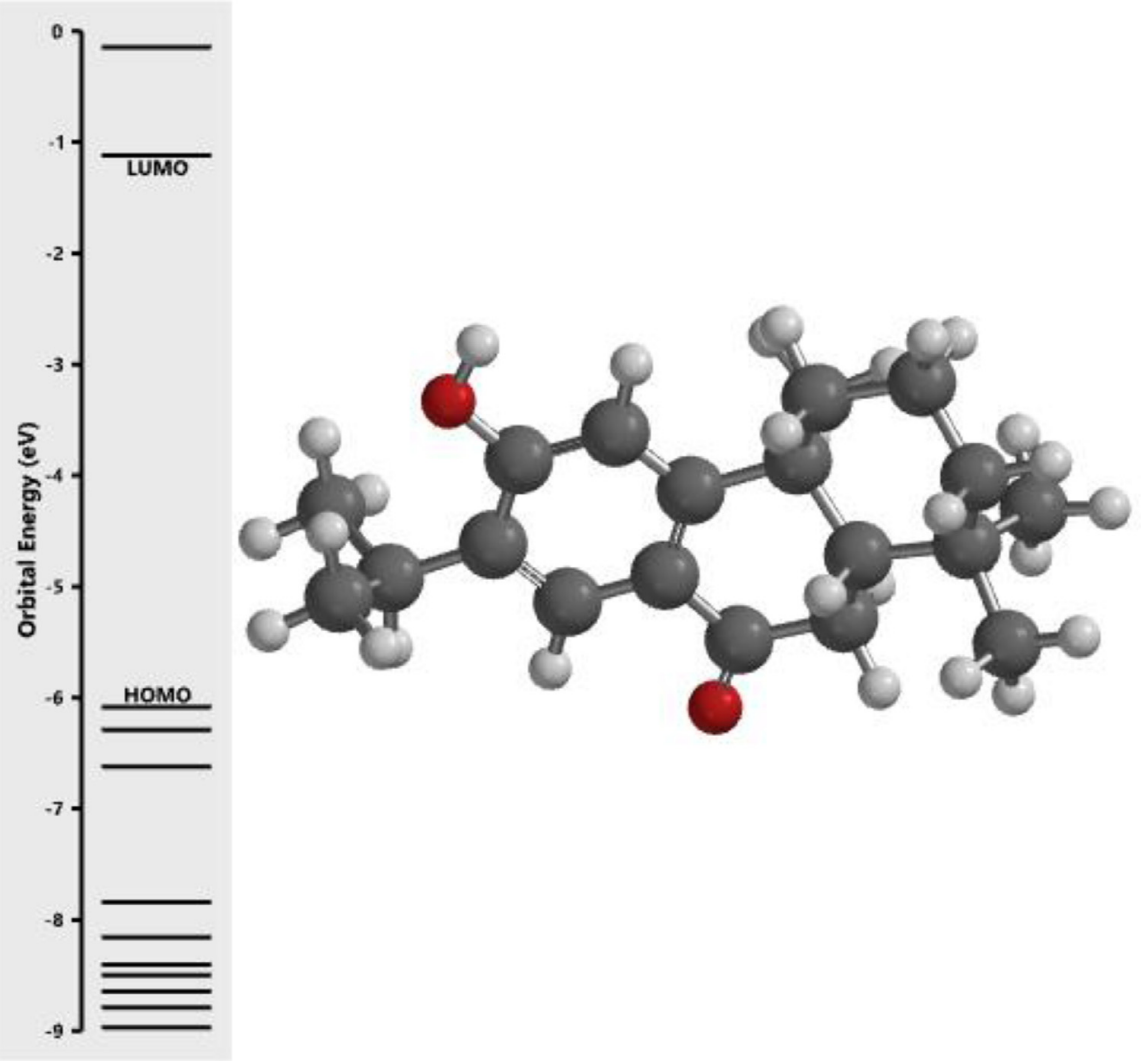
Predicted orbital energy for compound 14.

**Fig. 15:**
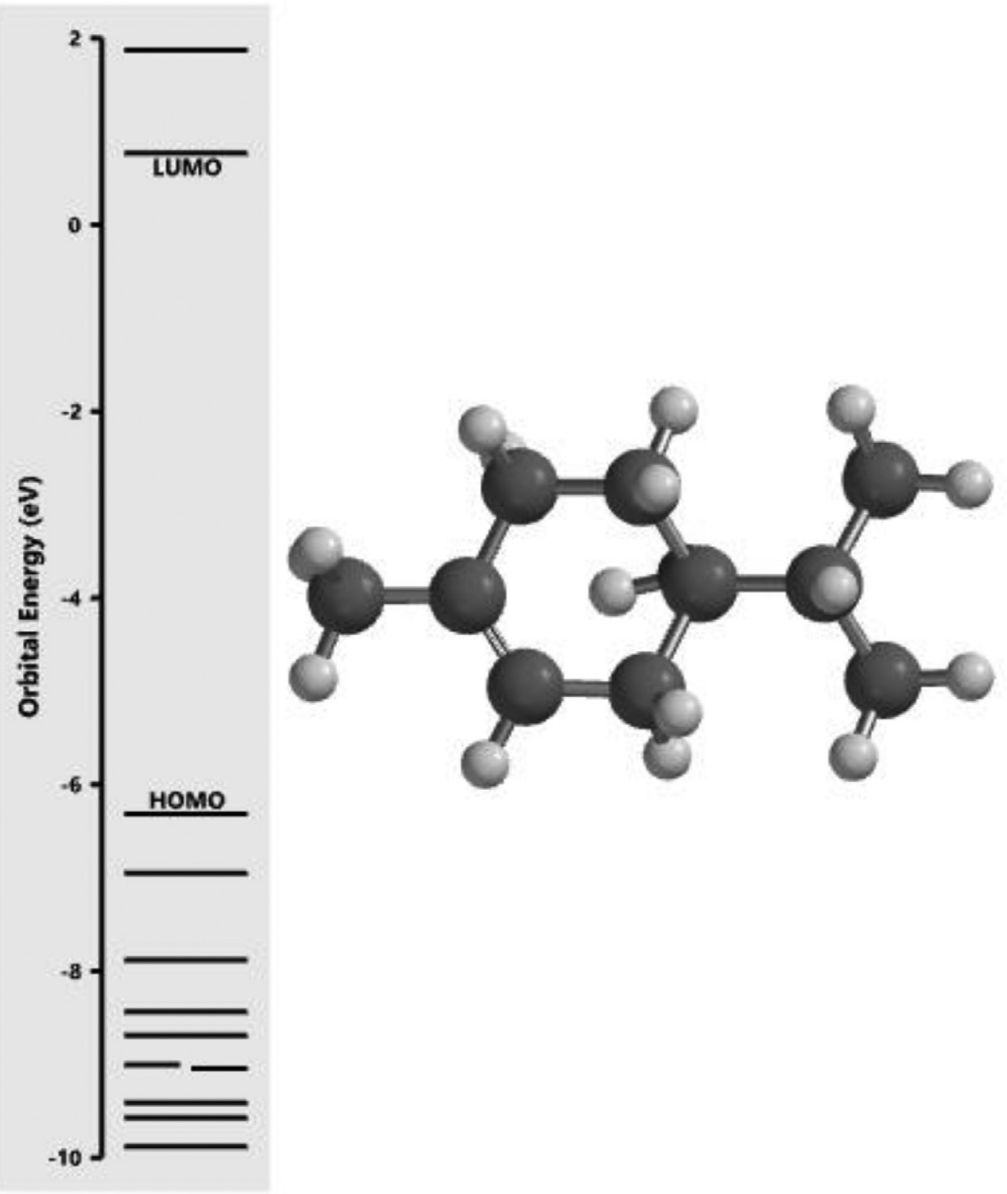
Predicted orbital energy for compound 15.

**Fig. 16:**
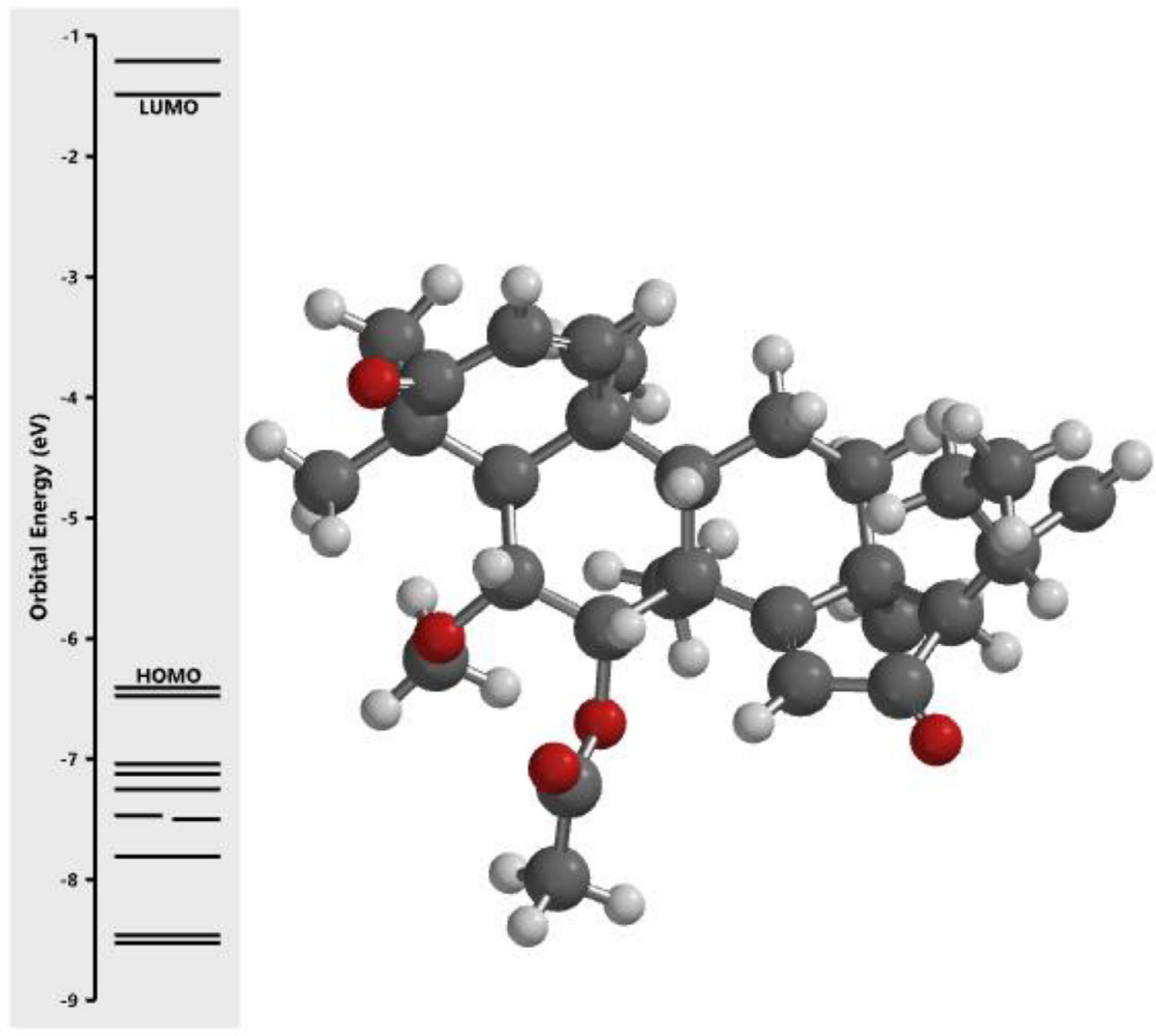
Predicted orbital energy for compound 16.

**Table 4:** HOMO-LUMO Overlay for the investigated phytochemicals.

	HOMO	LUMO
1		
		
2		
		
3		
		
4		
		
5		
		
6		
		
7		
		
8		
		
9		
		
10		
		
11		
		
12		
		
13		
		
14		
		
15		
		
16		
		

Table 5 revealed the statistical model developed as well as the factors considered for QSAR model validation using descriptors obtained from 3D structures of the selected phytochemicals via material studio software [2] (Table 6). The factors considered for reliability and validity of quantitative structure activity relationship (QSAR) model were squared correlation coefficient (R^2^), Adjusted R-squared, cross validation R-squared, significance of regression F-value, critical SOR F-value (95%) and Friedman LOF and the calculated value for each of the factor was 0.78, 0.70, 0.60, 10.64, 4.02 and 2.26 respectively. Also, the same factors were considered for the developed QSAR model using descriptors from 2D structures of the selected phytochemicals and the calculated value was 0.74 (squared correlation coefficient (R^2^)), 0.66 (Adjusted R-squared), 0.58 (cross validation R-squared), 8.92 (significance of regression F-value), 4.02 (critical SOR F-value (95%)) and 2.58 (Friedman LOF). the calculated and predicted Binding affinity were displayed in Table 7.

**Table 5:** Developed QSAR model via 3D descriptors.

Equation	R-squared	Adjusted R-squared	Cross validated R-squared	Significant Regression	Significance-of-regression F-value	Critical SOR F-value (95%)	Friedman LOF
IC_50_ = 0.120705013 (Area) - 0.083364691 (Volume) - 34.518560361 (Ovality) + 30.981589527	0.76	0.70	0.67	Yes	12.99	3.65	1.88

**Table 6:** Developed QSAR model via 2D descriptors.

Equation	R-squared	Adjusted R-squared	Cross validated R-squared	Significant Regression	Significance-of-regression F-value	Critical SOR F-value (95%)	Friedman LOF
IC_50_ = - 1.415485303 (nHeavyatom) + 0.011588189 (ATS1m) - 0.002624012 (ATS2m) - 3.830295651	0.74	0.66	0.58	Yes	8.92	4.02	2.58

**Table 7:** Calculated and predicted Binding affinity.

	Predicted Binding Affinity (3D Descriptors) (kcal/mol)	Predicted Binding Affinity (2D Descriptors) (kcal/mol)	Calculated Binding Affinity (kcal/mol)
1	−4.62938500	−6.52664	−4.9
2	−7.08445000	−4.8839	−7.2
3	−4.48506000	−4.73571	−4.9
4	−7.18887100	−6.51609	−7.1
5	−7.14426100	−7.69674	−7
6	−7.76795300	−7.62005	−7.8
7	−5.71832400	−4.02043	−3.8
8	−6.83798500	−6.15431	−6.8
9	−6.95996300	−6.84747	−6.8
10	−6.95996300	−6.66428	−7.1
11	−7.05438100	−6.66428	−7.1
12	−7.13565500	−7.75449	−7.7
13	−7.08833700	−6.13114	−6.8
14	−6.28523900	−6.41483	−7.1
15	−4.59304000	−5.20788	−4.7
16	−7.06713100	−7.42222	−7.2

**Fig. 17:**
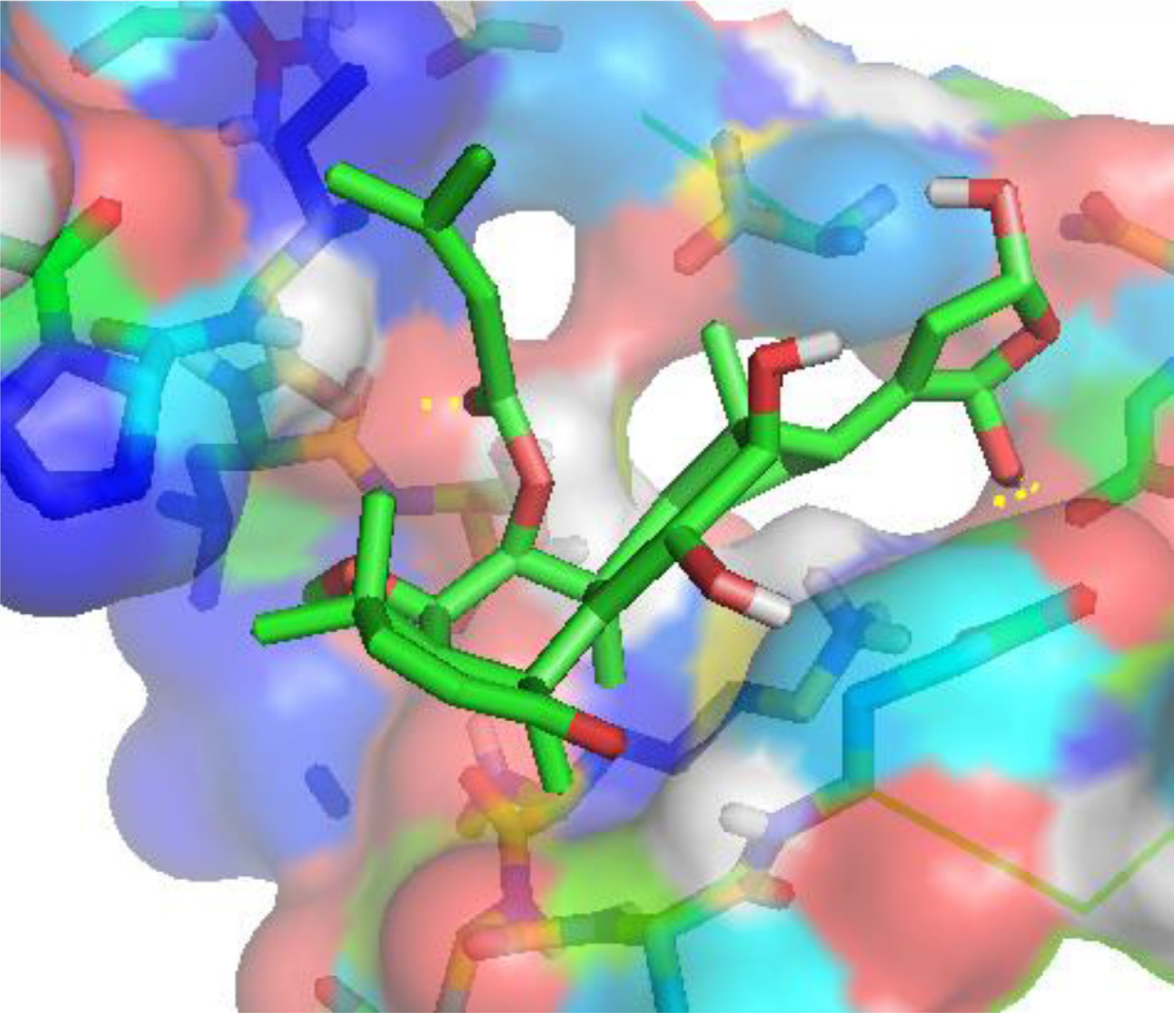
Pictorial image of docked compound 6 in the active site of NS2B–NS3 Proteases (PDB ID: 2fom).

The calculated binding affinity, residues involved in the interactions and types of non-bonding existing between the investigated complexes were shown in Table 8. The calculated binding affinity between the selected phytochemicals and NS2B/NS3 Protease (PDB ID: 2fom) [3] were −4.9 kcal/mol, −7.2 kcal/mol, −4.9 kcal/mol, −7.1 kcal/mol, −7.0 kcal/mol, −7.8 kcal/mol, −3.8 kcal/mol, −6.8 kcal/mol, −6.8 kcal/mol, −7.1 kcal/mol, −7.1 kcal/mol, −7.7 kcal/mol, −6.8 kcal/mol, −7.1 kcal/mol, −4.7 kcal/mol, and −7.2 kcal/mol for compound 1 - 16. The residue and types of interactions involved in the docked complexes were shown in Table 8 and Fig. 17.

**Table 8:** Calculated binding affinity and residues involved in the interaction.

	Binding Affinity (kcal/mol)	Residues involved in the interactions	Types of Non-bonding interaction
1	−4.9	Ala65, Trp61	Pi-Pi Stacked, Pi-Alkyl
2	−7.2	Ala56, Pro72,	Carbon Hydrogen Bond, Alkyl
3	−4.9	Ala65, Trp61	Pi-Pi Stacked, Pi-Alkyl
4	−7.1	Ile86, Leu74, Lys87, Pro72, Ile73, Ser75	Conventional Hydrogen bond, Carbon Hydrogen Bond, Alkyl
5	−7.0	Val59, Ala57, Ile73, Lys87, Ser75	Conventional Hydrogen Bond, Alkyl
6	−7.8	Glu54, Ile73, Lys87, Pro72	Conventional Hydrogen Bond, Carbon Hydrogen Bond, Alkyl
7	−3.8	Lys87, Ala56	Alkyl, Pi-Alkyl
8	−6.8	Ile86, Lys87, Ser75, Pro72	Conventional Hydrogen Bond, Pi-Alkyl, Alkyl
9	−6.8	Leu74, Pro72, Gln64	Conventional Hydrogen Bond, Alkyl
10	−7.1	Ala56, Pro72, Lys87	Conventional Hydrogen Bond, Carbon Hydrogen Bond, Alkyl, Pi-Alkyl
11	−7.1	Ala56, Pro72, Lys87	Conventional Hydrogen Bond, Carbon Hydrogen Bond, Alkyl, Pi-Alkyl
12	−7.7	Ser75, Leu74, Ile74, Pro72	Conventional Hydrogen Bond, Alkyl
13	−6.8	Ile73, Lys87, Pro72	Alkyl
14	−7.1	Ile86, Leu74, Pro72	Alkyl, Pi-Alkyl
15	−4.7	Glu62, Ala65, Trp61, Ile73	Conventional Hydrogen bond, Pi-Sigma, Alkyl
16	−7.2	Pro72, Asn88, Ile73, Ser75	Conventional Hydrogen bond, Carbon hydrogen bond, Alkyl
Metoclopramide	−4.7	–	–

Compound 6 and 12 with −7.8 kcal/mol and −7.7 kcal/mol were subjected to ADMET investigation via online admetsar2. The factors considered were for this work were blood-brain barrier (BBB), human intestinal absorption (HIA), Caco-2 permeability, p-glycoprotein substrate, p-glycoprotein inhibitor, renal organic cation transporter, subcellular localization, CYP450 2C9 Substrate, CYP450 2D6 Substrate, CYP450 3A4 Substrate, CYP450 1A2 Inhibitor, CYP450 2C9 Inhibitor, CYP450 2D6 Inhibitor, CYP450 2C19 Inhibitor, CYP450 3A4 Inhibitor, CYP Inhibitory Promiscuity, Human Ether-a-go-go-Related Gene, Inhibition, AMES Toxicity, Carcinogens, Fish Toxicity, Tetrahymena Pyriformis Toxicity, Honey Bee Toxicity, Biodegradation, Acute Oral Toxicity, Carcinogenicity (Three-class), Aqueous solubility, Caco-2 Permeability, Rat Acute Toxicity, Fish Toxicity, Tetrahymena Pyriformis Toxicity (Tables 9 and 10).

**Table 9:** Calculated ADMET properties for compound 6.

Model	Result	Probability
Absorption		

Blood-Brain Barrier	BBB-	0.7826
Human Intestinal Absorption	HIA+	0.9172
Caco-2 Permeability	Caco2-	0.6832
P-glycoprotein Substrate	Substrate	0.7726
P-glycoprotein Inhibitor	Inhibitor	0.6367
	Non-inhibitor	0.5595
Renal Organic Cation Transporter	Non-inhibitor	0.9196

Distribution		

Subcellular localization	Mitochondria	0.7278

Metabolism		

CYP450 2C9 Substrate	Non-substrate	0.8307
CYP450 2D6 Substrate	Non-substrate	0.8822
CYP450 3A4 Substrate	Substrate	0.6980
CYP450 1A2 Inhibitor	Non-inhibitor	0.8633
CYP450 2C9 Inhibitor	Non-inhibitor	0.7364
CYP450 2D6 Inhibitor	Non-inhibitor	0.9056
CYP450 2C19 Inhibitor	Non-inhibitor	0.8147
CYP450 3A4 Inhibitor	Inhibitor	0.5577
CYP Inhibitory Promiscuity	Low CYP Inhibitory Promiscuity	0.7672

Excretion		
Toxicity		

Human Ether-a-go-go-Related Gene Inhibition	Weak inhibitor	0.9943
	Non-inhibitor	0.7564
AMES Toxicity	Non AMES toxic	0.6613
Carcinogens	Non-carcinogens	0.9520
Fish Toxicity	High FHMT	0.9857
Tetrahymena Pyriformis Toxicity	High TPT	0.9904
Honey Bee Toxicity	High HBT	0.8420
Biodegradation	Not ready biodegradable	0.9788
Acute Oral Toxicity	III	0.3974
Carcinogenicity (Three-class)	Danger	0.4805

ADMET Predicted Profile — Regression		

Model	Value	Unit

Absorption		

Aqueous solubility	−3.0193	LogS
Caco-2 Permeability	−0.0240	LogPapp, cm/s

Distribution		
Metabolism		
Excretion		
Toxicity		

Rat Acute Toxicity	3.1592	LD50, mol/kg
Fish Toxicity	0.7960	pLC50, mg/L
Tetrahymena Pyriformis Toxicity	0.7365	pIGC50, ug/L

**Table 10:** Calculated ADMET properties for compound 12.

Model	Result	Probability
Absorption		

Blood-Brain Barrier	BBB+	0.7235
Human Intestinal Absorption	HIA+	0.9940
Caco-2 Permeability	Caco2+	0.6164
P-glycoprotein Substrate	Substrate	0.7957
P-glycoprotein Inhibitor	Non-inhibitor	0.5926
	Non-inhibitor	0.5739
Renal Organic Cation Transporter	Non-inhibitor	0.8069

Distribution		

Subcellular localization	Mitochondria	0.6990

Metabolism		

CYP450 2C9 Substrate	Non-substrate	0.8370
CYP450 2D6 Substrate	Non-substrate	0.8623
CYP450 3A4 Substrate	Substrate	0.7525
CYP450 1A2 Inhibitor	Non-inhibitor	0.5500
CYP450 2C9 Inhibitor	Non-inhibitor	0.8491
CYP450 2D6 Inhibitor	Non-inhibitor	0.9375
CYP450 2C19 Inhibitor	Non-inhibitor	0.8793
CYP450 3A4 Inhibitor	Non-inhibitor	0.8309
CYP Inhibitory Promiscuity	Low CYP Inhibitory Promiscuity	0.8842

Excretion		
Toxicity		

Human Ether-a-go-go-Related Gene Inhibition	Weak inhibitor	0.9464
	Non-inhibitor	0.7052
AMES Toxicity	Non AMES toxic	0.8217
Carcinogens	Non-carcinogens	0.9441
Fish Toxicity	High FHMT	0.9971
Tetrahymena Pyriformis Toxicity	High TPT	0.9942
Honey Bee Toxicity	High HBT	0.8392
Biodegradation	Not ready biodegradable	0.9850
Acute Oral Toxicity	III	0.4299
Carcinogenicity (Three-class)	Non-required	0.4859

ADMET Predicted Profile — Regression		

Model	Value	Unit

Absorption		

Aqueous solubility	−3.9102	LogS
Caco-2 Permeability	0.9842	LogPapp, cm/s

Distribution		
Metabolism		
Excretion		
Toxicity		

Rat Acute Toxicity	2.5657	LD50, mol/kg
Fish Toxicity	0.3250	pLC50, mg/L
Tetrahymena Pyriformis Toxicity	0.8839	pIGC50, ug/L

## 2 Experimental Design, Materials and Methods

**Fig. 18:**
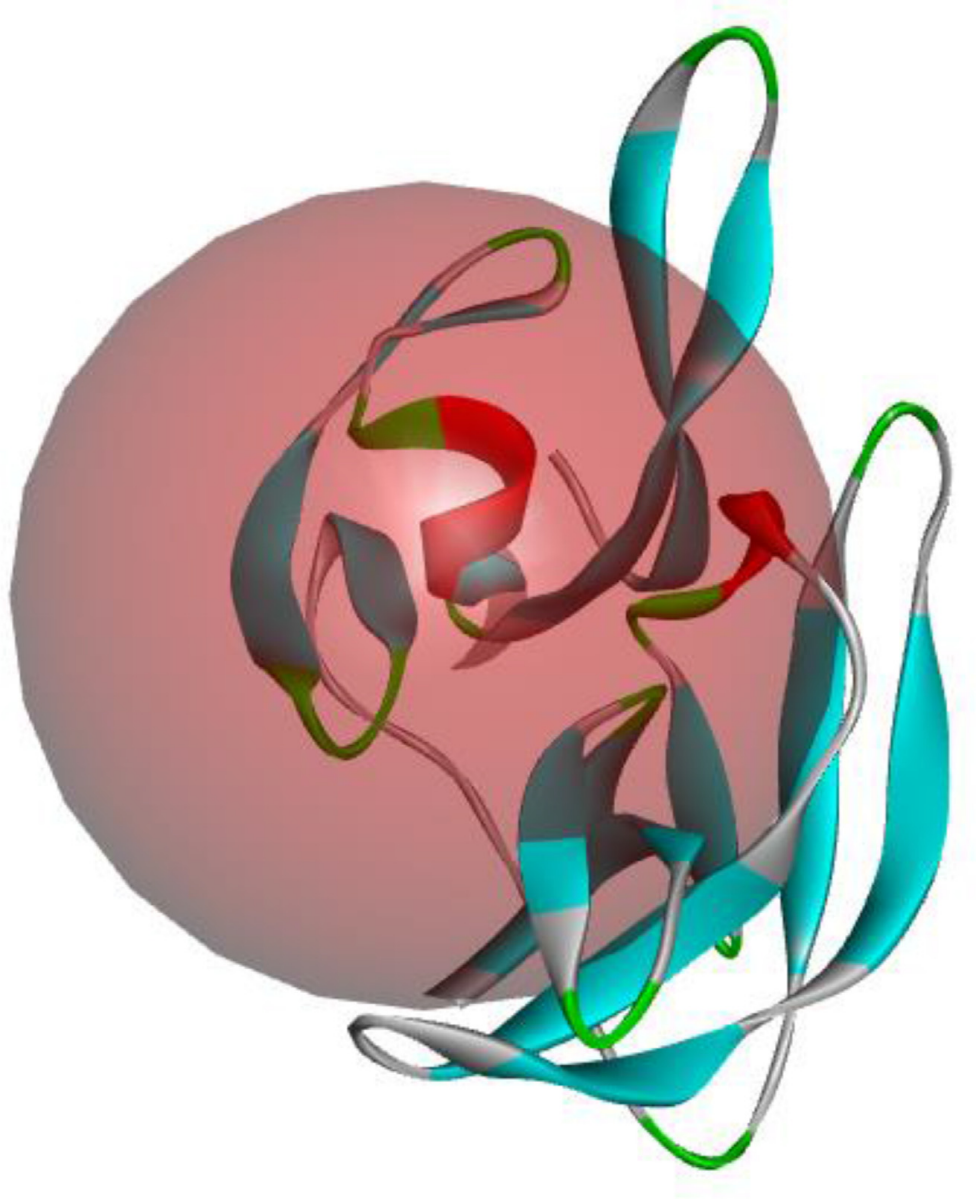
3-Dimensional structure of NS2B–NS3 Proteases with identified binding site.

Sixteen phytochemicals in *Azadirachta indica*. L were selected from the work done by Saleem et al. [1] which were subjected to optimization using 6-31G** as basis set via Spartan’14 tool [4,5]. The selected phytochemicals were optimized in solvent (water) so as to mimic the environment of therapeutic agent in human being and the duration for completion of individual compound depends on the content of the compound as well as the chosen basis set. The descriptors obtained from 2D and 3D structures of the selected compounds were subjected to QSAR analysis using material studio software [6] and the predicted binding affinity was reported. Also, the developed QSAR models were validated in order to ascertain the reliability of the models; thus, Adjusted R-squared, cross validation R-squared, significance of regression F-value, critical SOR F-value (95%) and Friedman LOF were considered. More so, the optimized compounds were subjected docking analysis and the binding affinity as well as interactions between the investigated complexes was observed. The factors considered for the choice of 2FOM as the downloaded receptor were resolution, R-value free, R-value work and R-value observed which were observed to be 1.50Ǻ, 0.209, 0.176 and 0.177 respectively. The receptor (NS2B/NS3 Protease (PDB ID: 2fom) [3]) used in this work was obtained from protein data bank which was treated using pymol software [7] before identifying the active site of the receptor using Autodock tool [7] and the value observed for the grid box were: center (*X* = 6.631, *Y* =−23.612, *Z* = 18.525) and size (*X* = 40, *Y* = 82, *Z* = 44); spacing was set to be 1.00 Å (Fig. 18). The docking calculation was executed on the prepared ligand and the receptor using autodock vina [8]. The compound with lowest binding affinity value was subjected to ADMET for further investigation.

## CRediT authorship contribution statement

**Abel Kolawole Oyebamiji:** Conceptualization, Methodology, Data curation, Writing – original draft, Visualization, Investigation, Writing – review & editing. **Sunday A. Akintelu:** Conceptualization, Methodology, Data curation, Writing – original draft, Visualization, Investigation, Writing – review & editing. **Ismail O. Akande:** Data curation, Visualization, Investigation. **Halleluyah O. Aworinde:** Methodology, Data curation, Writing – original draft. **Oluwafunmilola A. Adepegba:** Data curation, Writing – review & editing. **Emmanuel T. Akintayo:** Methodology, Data curation, Writing – original draft. **Cecillia O. Akintayo:** Methodology, Data curation, Writing – review & editing. **Banjo Semire:** Writing – original draft, Supervision, Writing – review & editing. **Jonathan O. Babalola:** Writing – original draft, Supervision, Writing – review & editing.

## Declaration of Competing Interest

The authors declare that they have no known competing financial interests or personal relationships which have or could be perceived to have influenced the work reported in this article.

## Data Availability

2D descriptors for selected compounds from Azadirachta indica L (Original data) (Mendeley Data).
